# The Aarhus statement on cancer diagnostic research: turning recommendations into new survey instruments

**DOI:** 10.1186/s12913-018-3476-0

**Published:** 2018-09-03

**Authors:** Domenica Coxon, Christine Campbell, Fiona M. Walter, Suzanne E. Scott, Richard D. Neal, Peter Vedsted, Jon Emery, Greg Rubin, William Hamilton, David Weller

**Affiliations:** 1 Centre for Population Health Sciences, The University of Edinburgh, Edinburgh, EH8 9DX UK; 2 Primary Care Unit, Department of Public Health and Primary Care, University of Cambridge, Cambridge, UK; 3 Dental Institute, King’s College London, London, UK; 4 Leeds Institute of Health Sciences, University of Leeds, Leeds, UK; 5 Research Unit for General Practice, Aarhus University, Aarhus, Denmark; 6 General Practice and Primary Care Academic Centre, University of Melbourne, Melbourne, Australia; 7 School of Medicine, Pharmacy and Health, Durham University, Stockton-on-Tees, UK; 8 Primary Care Diagnostics, Peninsula College of Medicine and Dentistry, Exeter, UK

## Abstract

**Background:**

Over recent years there has been a growth in cancer early diagnosis (ED) research, which requires valid measurement of routes to diagnosis and diagnostic intervals. The Aarhus Statement, published in 2012, provided methodological guidance to generate valid data on these key pre-diagnostic measures. However, there is still a wide variety of measuring instruments of varying quality in published research. In this paper we test comprehension of self-completion ED questionnaire items, based on Aarhus Statement guidance, and seek input from patients, GPs and ED researchers to refine these questions.

**Methods:**

We used personal interviews and consensus approaches to generate draft ED questionnaire items, then a combination of focus groups and telephone interviews to test comprehension and obtain feedback. A framework analysis approach was used, to identify themes and potential refinements to the items.

**Results:**

We found that many of the questionnaire items still prompted uncertainty in respondents, in both routes to diagnosis and diagnostic interval measurement. Uncertainty was greatest in the context of multiple or vague symptoms, and potentially ambiguous time-points (such as ‘date of referral’).

**Conclusions:**

There are limits on the validity of self-completion questionnaire responses, and refinements to the wording of questions may not be able to completely overcome these limitations. It’s important that ED researchers use the best identifiable measuring instruments, but accommodate inevitable uncertainty in the interpretation of their results. Every effort should be made to increase clarity of questions and responses, and use of two or more data sources should be considered.

## Background

In the UK poor survival from cancer is attributed largely to late stage diagnosis; there is growing evidence that diagnostic intervals influence prognostic outcomes [1–3]. Describing, in a systematic and valid way, the contribution of routes to diagnosis, and associated diagnostic intervals, to survival difference between countries is a current priority for the international research community – an example of this effort is the International Cancer Benchmarking Partnership [4].

**Fig. 1:**
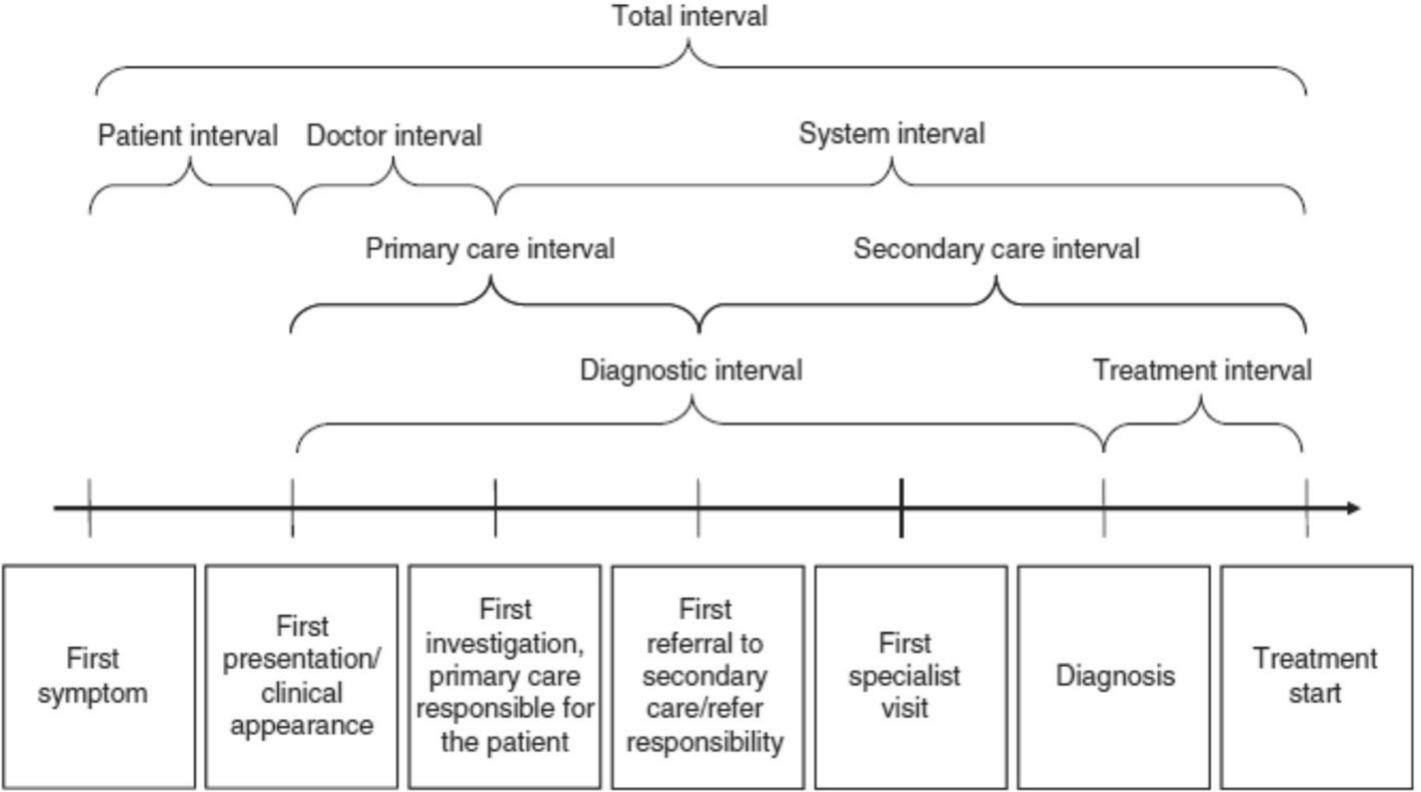
Cancer diagnostic intervals used in developing the Aarhus Statement [19]

The lack of consensus on the definitions and methods used in cancer diagnosis research led to the development and publication of the Aarhus statement [5], which provides methodological guidance on recording and reporting of time-point measurement, with the intention of promoting consistency in studies based on diagnostic intervals [6]. It also provides recommended definitions of the key time-points for diagnostic and treatment pathways - from date of first presentation through to diagnosis and treatment (see Fig. 1). For example, the ‘date of first presentation’ is defined as ‘*the time point at which, given the presenting signs, symptoms, history and other risk factors, it would be at least possible for the clinician seeing the patient to have started investigation or referral for possible important pathology, including cancer*’.

However, to date little work has explored how questionnaire/survey items, based on the Aarhus Statement might be interpreted by patients and their health care providers - and the extent to which responses are a true reflection of the measured time-points [7, 8].

Accordingly, in this study we developed a series of survey items about time-points, intervals and routes to diagnosis, based on Aarhus Statement definitions, with the aim of testing the items with patients with two common cancers (colorectal and lung cancer), GPs and cancer diagnosis researchers (CDRs). The key elements of the diagnostic pathway we examined are shown in Table 1. As described in the Aarhus Statement [5]; 1) there are multiple *routes to diagnosis* for cancer patients, including presentation to a GP with symptoms, emergency admissions and screening. Better understanding of the pattern of diagnostic routes in a population can help in efforts to improve patient pathways 2) the *key symptom prompting action* and 3) *date of first presentation* are also vital, but can be hard to define, particularly in the presence of longstanding or multiple symptoms 4) the volume/extent of pre-diagnostic activity is important, as it reflects the level of burden on patients, costs and complexity of patient pathways 5) *date of referral* helps define the primary care interval, but can cause confusion; particularly if there is lack of clarity over what the referral is for (eg investigations, specialist consultations etc), and 6) date of diagnosis, the end-point of the diagnostic pathway, can be defined in various ways – and patients and health care providers can differ in their perceptions of this date.

**Table 1:** Diagnostic pathway elements, survey items generated from Aarhus Statement definitions, and participant responses

Element of diagnostic pathway	Survey items generated	Exemplar participant responses
Route to diagnosis (eg through visit to GP, via accident and emergency etc)	Which of the following best describes the events which led to your diagnosis of cancer? • I had symptoms/I noticed a bodily change and went to see a doctor (eg GP, family doctor) • I had symptoms/I noticed a bodily change and was taken to Accident and Emergency • I was being investigated by my doctor(s) for another problem during which time the cancer was discovered • I had a cancer screening test (eg as part of a programme offered by a health professional)	*interviewer: OK so you don’t think you would fit it either of these?* *‘well what he’s saying he went to A&E but he wasn’t diagnosed there’* *(colorectal patient)* *‘there’s sometimes a bit of overlap, so I think for the majority of people those four options are appropriate. There are times when someone might have symptoms, but then attend A&E’ (GP)* *I suppose if you were to add, I was being investigated or monitored by my doctor for another problem during which the cancer was discovered, then that covers it’ (CDR)* *what are we talking about here? Are we trying to satisfy the sociologists? That’s not the purpose. I wouldn’t use anything like ‘bodily change’; I would use words, proper language, like ‘something was wrong with me’ (CDR)*
The key symptom/health problem which prompted action (eg visiting a doctor) by patient	• Looking back to the events which led to your diagnosis of cancer, what was the main health problem or symptom that made you contact a doctor? • Can you recall any other symptoms or health problems which you now believe were associated with the cancer?	*‘I think that’s a very difficult question, sometimes it’s easy, mine was passing blood, other times it could be, as you say, a subtle thing that you don’t realise’ (colorectal patient)* *‘So for alarm symptoms, I think it’s easier. But we know that most cancers don’t actually present with alarms symptoms, so I think for those cancers that don’t present with an alarm symptom, then it…then I think it is a bit more problematic’ (GP)*
Date of first presentation to primary care	• When did you first go to your general practitioner with your symptoms of cancer? • When did you first seek help for the symptoms which you now believe were caused by your cancer?	*In response to a patient scenario: ‘I think I would go for January 2008 on the grounds that the cough was somehow kind of different and that the cough had changed, so he might have had a kind of smoker’s cough before but it was different for January 2008’ (lung patient)* *‘one of the difficulties is….. where you’ve got somebody with a chronic recurring problem going on for years, you know, without cancer being, you know, maybe at the very first presentation cancer being an issue, because of the cough or the diarrhoea or abdominal pain, but then once it’s excluded, you say, no, you’ve got irritable bowel syndrome or, you know, you’ve got COPD and then these patients come back time and time again and then these two incidences that you’ve documented, you know, they’re people with the same symptoms for eight, 10 years….it’s the horrible difficulty that we have in primary care saying, right, when is this exacerbation of COPD or an exacerbation of irritable bowel syndrome’ (GP)*
Volume/extent of pre-diagnostic activity	• How many times did you visit a GP/primary care physician/family doctor and or hospital about your symptoms before your cancer was diagnosed? • How many different doctors did you see in the lead up to your diagnosis? • How many different doctors/hospital departments did you visit in the lead up to your cancer diagnosis?	*‘it probably also depends on the complexity of their investigation. Some will be going quite easily through a fast track pathway and others they will…and of course it’s those patients that we want to be able to make a distinction between. Would it be easier to have boxes they can tick off, two, four, six?’ (CDR)* *‘It’s a very fluid thing actually, so a lot of the…and a lot of stuff happens to…at the same time and in different…I find that really difficult to make a clear distinction between one and the other’ (GP)* *‘maybe you need to say how many times you’ve been to the GP, how many times, how many hospital visits did you have before the cancer diagnosis, separate into two questions (colorectal patient)*
Date of referral	• When did your GP refer you to a specialist to investigate you for symptoms of cancer? • When would you say the responsibility for further diagnosis and management of your cancer transferred from your GP to specialist cancer services?	*‘the GP may refer somebody for investigation it might be a x-ray or a CT scan but if you were saying referred to a specialist that comes after the GP has got the results of the x-ray or the scan, so in a sense there’s two things, there’s one referral for investigation which might not come to anything and then there’s referral to the specialist, so I think we just need to be clear which one we’re looking for’ (lung patient)* *‘I just thought it was another process, the thing that I was going through and I thought they all worked together and my GP was there to guide me to these people eh, but I could still go back and see my GP whenever I wanted, do you know what I mean, so it wasn’t like he cut off from, it was not left to the specialist, you still have to visit your GP and that, not as much obviously, but he was always there to talk as well if I wanted’ (colorectal patient)* *‘it might go back to the definition of referral or how it’s understood, because I suppose it seems that your understanding is not that the responsibility is gone forever and the patient has been completely handed over because you’ll still be involved in their care. Which is why I think the word transferred we’ve used here is wrong’ (GP)*
Date of diagnosis	• When was your cancer diagnosed? • What are you basing this date on? (eg date you were told, date of operation, date of tests etc)	*‘Yeah I mean it’s difficult because is somebody saying to you I’m 90% sure that this is malignant, it’s cancer, is that a confirmed diagnosis or was it the next week when that same person rang me up and he said ah we’ve had the result of the biopsy and I was right, so from my point of view I would say it was when he told me he was 90% sure, because for me that’s a fairly strong indication’ (colorectal patient)* *‘I would say for me as well the fact that it was the consultant that did my colonoscopy, that was when I found out that I had cancer, not when I went to see the oncologist or the surgeon or anything, they confirmed it obviously but it’s the, I would say it’s the first person, I suppose like you were saying the first person that mentions the c word’ (colorectal patient)* *‘You never forget the date, you remember the date he told you you had cancer’ (lung patient)* *‘are you meaning at what stage did the patient understand the diagnosis, rather than when was the GP told by the hospital, this patient has got X wrong with him? They’re not simultaneous’ (non-cancer patient)* *‘you could argue than when the bronchoscope actually looks at the…eyeballs the tumour, they’re able to say, you’ve got cancer, but I think most people would wait for the histology’ (GP)*

## Methods

We firstly generated questionnaire/survey items derived from Aarhus Statement definitions (see Table 1) – item generation was undertaken through a series of personal interviews with early cancer diagnosis researchers and clinicians (followed by a group consensus process, and an examination of wording in existing validated instruments [1, 9], drawing particularly on questionnaires used in the International Cancer Benchmarking Partnership ‘Module 4’ surveys) [4]., We sought items which related to 1) routes to diagnosis: for example, did patients see a GP first, go straight to an A&E department etc., 2) the symptom(s) and associated characteristics which prompted help-seeking activity and 3) key time-points and intervals, such as date of first presentation and date of referral – we prompted the researchers and clinicians to take account of the complexity in defining some of these time-points. We were particularly interested in items which had high relevance to early diagnosis, avoided abstract terms and jargon, provided sufficient clarifying details and included a spectrum of response options; in general we favoured open-ended over closed questions. We then used a combination of focus groups and telephone interviews with patients (with or without a cancer diagnosis), GPs and cancer diagnosis researchers (CDRs), conducted in May and June 2015 in Edinburgh, UK to respond to the draft items. There were both ‘patient’ and ‘GP’ versions of the items (only the patient questions are shown, but the wording of the GP questions was very similar).

### Recruitment

Cancer patients were recruited either through the Edinburgh Cancer Centre or their general practice. Cancer Centre patients were identified by a clinician (nurse or consultant) and invited by the study researcher to participate in the study; assurance was required from the treating clinician that the patient had the ability to participate in focus group discussions and capacity to provide informed consent. Thirteen patients with colorectal cancer and 12 with lung cancer, all diagnosed in the previous 9 months, were recruited; there were no age restrictions. Eighteen participants without cancer were recruited from two general practices in Edinburgh (one each in areas of high and low deprivation). GPs and CDRs were identified based on existing UK and international professional networks and contacted via email to participate in the study. GPs (*n* = 4, 3 M, 1F) were based in Scotland and associated with university departments of general practice. Cancer diagnosis researchers (*n* = 6, 5F, 1 M) were based in Scotland, England, Australia, Canada and Denmark; they were identified through existing UK and international cancer research networks; their disciplinary expertise included general practice, epidemiology, social science and psychology - all had at least 10 years of experience in early cancer diagnosis research.

### Focus groups – patient participants

The focus groups were semi-structured, and conducted separately for colorectal, lung and non-cancer patients by the study researcher (DC). They took place either at the Edinburgh Cancer Centre or the participants’ general practice. Participants were presented with the background and rationale for the study, and the Aarhus Statement-derived survey items. Participants were asked to identify, for each item, the potential challenges they might face as patients in comprehending the item and articulating responses. Participants were asked how ‘easy’ or ‘difficult’ they might find it to identify the key time-points in their own pathway to diagnosis. The focus group guide incorporated a series of probes based on the cognitive model for survey response [10]. These probes were:

- *comprehension*: what the respondent believed the question to be asking, what specific words and phrases meant to the respondent;
- *recall*: the type of information that the respondent needed to recall in order to answer the question;
- *judgment:* whether the respondent devoted sufficient mental effort to answer accurately and thoughtfully;
- r*esponse-mapping:* whether the respondent matched his or her internally generated answer to the response categories given by the survey question

To further test comprehension, participants were presented with a hypothetical patient story involving a complex patient pathway and asked for the dates which they considered best reflected the time-point under consideration. Participants were asked for further views about the proposed approaches to establishing these time-points in surveys of cancer patients.

### Telephone interviews – GPs and CDRs

Interview documentation was sent to GPs and CDRs in advance via email, and a convenient time arranged. The telephone interviews were semi-structured; GPs were asked for their views on their own challenges, and those they anticipated their patients would face, in providing responses to the survey items. CDRs were asked how they felt patients and GPs would respond to the items – and invited to comment on the likely validity of the survey item responses. Again, participants were presented with a series of hypothetical patient stories to help elucidate issues in identifying time-points in complex diagnostic journeys. As with the patient interviews, the interview schedule incorporated probes based on the cognitive model for survey response [10].

All patient participants were offered £15 incentive vouchers and compensation for their time and travel expenses.

### Data analysis

All focus groups and telephone interviews were audio-recorded, transcribed verbatim and analysed using framework analysis [11]; we chose this approach as it is both pragmatic and iterative, with the capacity to respond to themes as they emerge while maintaining a clear link to the original transcripts. Two researchers (DC and SS) developed an agreed framework involving a ‘double indexing’ approach - both researchers applied the framework separately to one transcript from each group. Once consensus in coding had been reached, DC applied the framework to the remaining focus group transcripts and indexed and charted the themes for each survey item. To increase credibility, another two researchers successfully applied the same framework to two transcripts to gain consensus about its suitability and applicability. During the mapping and interpretation phase, implications for the development of the items were considered by all members of the project team.

### Ethical approvals

The study protocol was approved by the local research ethics committee: South East Scotland (Ref: 13/SS/0221)**.**

## Results

### Participant characteristics

Participant characteristics are summarised in Table 2. Patients had a mixture of co-morbidities including arthritis, carpel tunnel syndrome, hypertension, atrial fibrillation, heart disease, diabetes, osteoporosis, circulatory and rheumatic problems. GPs (*n* = 4) were based in Scotland; 3 were male. CDRs (*n* = 6) were based in Scotland, England, Australia, Canada and Denmark; 5 were female. The survey items and the responses they generated, guided by Aarhus Statement definitions are shown in Table 1. We divided responses into the 6 previously described elements of the diagnostic pathway.

**Table 2:** Focus group participant characteristics

	n	Age range (median)	Sex	Employment status
Colorectal cancer	13	52–91 (67)	M 10 F 3	4 employed, 6 not working due to ill health, 3 retired
Lung cancer	12	43–89 (65)	M 8 F 4	8 employed, 2 not working due to ill health, 2 retired
Non-cancer	18	54–90 (75)	M 12 F 6	12 employed, 6 retired

### Route to diagnosis

Responses (shown in Table 1) reflect the complexity of pre-diagnostic pathways, with many participants indicating the 4 response categories didn’t cover all options – for example if there had been a series of GP and A&E visits in the lead up to the diagnosis. GP respondents also suggested that there are overlaps between the potential pathways to diagnosis and that some patients will have a combination of two. CDRs queried whether the first pathways to diagnosis options cover *incidental* discoveries (for example, a potential indicator of malignancy in a routine blood test) and whether a patient would think they’d been under ‘investigation’ by a doctor in this case. Both cancer and non-cancer patients indicated they understood the difference between screening and symptom-based (or incidental) cancer detection, but raised the issue that many subsequent questions were not relevant to patients who have had their cancer detected via screening programmes. GPs were less sure that patients always understood the difference between screening and symptom-based diagnosis, particularly when investigations, such as a faecal occult blood test, could be used as both a ‘screening’ and a ‘diagnostic’ test.

There were also concerns about terminology; the term ‘bodily change’ caused some confusion amongst participants, as many symptoms don’t produce changes in the body such as lumps or weight loss. Others felt that symptoms such as ‘rectal bleeding’ were, indeed, bodily changes. CDRs suggested a greater use of lay language.

### Main symptom/health problem which prompted action

There was consensus amongst almost all respondents that symptoms can be difficult to recall – and may not be thoroughly recorded in GP notes. Identifying a ‘main symptom’ was considered to be potentially difficult, particularly in the context of multiple or vague symptoms. Similarly, GPs found it easier to identify the main presenting symptom when it was an ‘alarm symptom’, rather than if the patient had multiple or vague symptoms. Again there were issues around terminology; one CDR thought the word ‘associated’ might invite respondents to consider environmental or other causes of cancer.

### Date of first presentation to primary care

As expected, participants differed in their understanding of this date, particularly in the presence of vague, multiple, intermittent or longstanding symptoms – there was no consensus on how far in the past people should attempt to recall symptoms. Many highlighted dates of consultations prompted by *changes* in longstanding symptoms (for example, when a cough became ‘hacking’ in nature). Moreover, in some cases there was a misattribution of symptoms to an existing disease (eg irritable bowel syndrome or COPD) making accurate identification of date of first presentation difficult. GPs found recall for date of presentation more difficult for patients who were frequent attenders, or had chronic diseases – patients with ‘multiple morbidities’ posed a particular challenge, as consultations were often complex, and symptoms of oncological significance could be masked by other symptoms or priorities.

### Volume/extent of pre-diagnostic activity

CDRs suggested that recall difficulty depends on complexity of pathways; they suggested that patients will remember a few consultations at most. GPs suggested that recall of *extent* and *nature* of activity is difficult, and the distinction between activity in primary versus secondary care can become blurred; they suggested listing the specialities of doctors seen (eg radiologist, GP, oncologist) and the types of investigations undertaken (eg chest x ray/CT scan/endoscopy/blood tests/fine needle aspiration etc). Indeed, most respondents suggested this question was ‘lumping a lot together’ and that it could be separated out, specifying ‘hospitals departments’ and ‘doctors/specialties’.

### Date of referral

This date was not always straightforward for respondents. The greatest source of confusion arose over the issue of what the referral was actually for – an investigation or a specialist consultation. Similarly cancer patients queried the meaning of ‘referral’: we had suggested it was the process by which ‘responsibility is transferred’ from their GP to a specialist, but many respondents indicated the responsibility would continue to be shared after the referral. GPs queried whether the term referral means a ‘transfer of care from the GP’ since the GP will continue to see and care for the patient thereafter.

### Date of diagnosis

Patient participants mostly considered that ‘diagnosis’ constitutes the date when the patient is first ‘told’ (often of a suspicion by a consultant or by a GP). One respondent argued that the pathology report is the official final diagnosis but the majority believed that pathology is a means of staging the extent of the cancer. Overall, though, cancer patients agreed that date of diagnosis (the date they were told) is considered so significant it can be recalled easily (particularly with the assistance of a diary) – so it is the date they are most likely to report. Non-cancer patients also emphasised that the clinical diagnosis (as perceived by a health care provider) is not the same as the patients’ experience of the diagnosis – this likely contributes to differences between doctor and patient reports of date of diagnosis. Most GPs indicated that they take the date of confirmation of histology as the date of diagnosis. It was suggested that receipt of the consultant’s letter/date GP informed (even though the GP may, at times, make the diagnosis) are also important and so should be included in response options.

## Discussion

Our study adds to a growing body of methodological literature on measurement of routes to a cancer diagnosis and diagnostic time intervals; it demonstrates the many challenges and pitfalls associated with their accurate measurement. We based our survey items as closely as possible on Aarhus Statement definitions, yet found that enquiry often still prompted considerable uncertainty. While there is growing interest in measuring cancer diagnostic intervals, to examine their effect on cancer outcomes and to make international comparisons, we currently lack standardised measuring instruments. The challenges in measurement of routes to diagnosis arise from the frequent complexity of cancer patients’ journeys, the existence of co-morbid conditions, and the nature of cancer symptoms, which are often multiple and vague [6, 12, 13].

### The importance of accurate measurement in ‘routes to diagnosis’ research

Establishing patterns of routes to diagnosis is important for health service planning – for example, there is current interest in reducing emergency cancer presentations [14, 15]; measuring outcomes of any initiatives in this area requires a clear distinction between a cancer diagnosed in general practice compared to a cancer diagnosis arising from a presentation to A&E. Our study demonstrates the care needed in making this distinction, because these two diagnostic paths can, in the presence of multiple presentations over a limited period of time, become blurred. Similarly, while our patient respondents denied any confusion over what constitutes a screening pathway (eg no symptoms, participation in an organised programme), other research has demonstrated that confusion does, indeed, often exist [4], and patients’ understanding of the route they’ve taken needs careful interpretation.

Defining the ‘main symptom’ which prompted help-seeking is important in early diagnosis research but, again, this symptom may be elusive – and, it may be influenced by pre-existing knowledge [6, 16]; or mis-attribution, particularly in the presence of chronic illness or multiple symptoms [17].

The primary care interval (beginning with date of first presentation and ending with date of referral) is also the subject of international interest; in systems where primary care acts as the ‘gatekeeper’ to health services, it’s important to ensure that symptoms are responded to in a timely and appropriate way, and referrals made to appropriate specialist cancer services. So we need accurate measures of this interval but, particularly in the presence of multiple or vague symptoms, which may be persistent for long periods of time, ‘date of first presentation’ can be difficult to specify. Even with the help of case records, GPs may struggle to identify at which consultation the ‘first’ presentation of a symptom occurred. Similarly, date of referral and date of diagnosis can cause confusion if not sufficiently specified.

### Study limitations and strengths

Our study confined its analysis to survey items which might be used in self-completion surveys. There are other methods for measuring diagnostic intervals and routes to diagnosis; the interactive dialogue of an interview-based survey would, for example, allow some of the comprehension and clarity issues of time-point recall to be resolved. But it is likely also that self-completion questionnaires will remain a feature of ‘routes to diagnosis’ cancer research, as they enable large sample sizes - so it is important to understand the cognitive processes respondents utilise, and the challenges in comprehension they face, when responding to written questions.

Focus group discussions can lead to bias - for example, participants may alter their view in order to fit into the group or as an attempt to please the researcher/facilitator (‘social desirability bias’) [18]. Nevertheless, the study used an experienced qualitative researcher and employed techniques to minimize this bias.

We’ve not formally tested the psychometric properties of the items we’ve identified – however, in the next phase of the study (reported separately) we undertake an e-delphi consensus process in which we further examine, for both patients and GPs, how *understandable* the items are, and how *feasible* respondents consider the items are to answer. Nevertheless, we acknowledge the limitations of self-completion instruments, and the constraints on their capacity to capture some of the complexity of cancer diagnostic journeys. We’ve purposefully not produced, as part of this work, an ‘off-the-shelf’ validated instrument which other CDRs might use. Rather, we considered that the most useful output would be to 1) see the questions we tested, 2) hear from potential recipients of ED questionnaires (principally patients and GPs) about the potential value and limitations of the questions, and 3) to produce a list of recommendations which might guide future instrument development.

Finally, even though we obtained broad and diverse responses from our GPs and CDRs, the number of participants was small, which places some limitations on the data.

### Recommendations

We recommend that self-completion-based surveys either include *extra clarifying text*, or *opportunities to directly interact with participants*, to address the following issues:

- *Route to diagnosis* – researchers should accommodate the uncertainty over emergency/A&E presentations versus diagnoses via primary care routes. There are scenarios (such as GP referral to A&E) which can lead to lack of clarity, and methodological guidance on this key issue is emerging [15]. Steps should also be taken to reduce ambiguity over what constitutes ‘screening’. While health care providers can readily identify a screening route to diagnosis, patients may not appreciate the difference between a screening and diagnostic examination – particularly, for example, if they had symptoms at the time of taking a screening test. There are also international differences in the ways terms such as ‘screening’ and ‘early diagnosis’ are interpreted

- *Main symptom prompting action* – there may not be single symptom – and the symptom(s) may be vague or intermittent. Indeed, one ‘main symptom’ may be replaced by another.

- *Date of first presentation* – diagnostic journeys are often non-linear, and a ‘true’ date of first presentation may be hidden amongst other pre-diagnostic activity. In some cases it may not be possible to identify a single consultation representing ‘first presentation’ – in which case a pragmatic but transparent approach might be needed to identify a ‘date of best fit’.

- *Extent of pre-diagnostic activity* – in many cases this can be too much to recall; ideally prompts should be used to categorise this activity

- *Date of referral* – there should be clarity over what the referral was for – the Aarhus statement recommends transfer of care to specialist services, rather than referral for diagnostic investigations [5]. In gatekeeper systems we seek to define this date as it represents the end of the primary care interval – but caution is needed; as our patient and GP respondents indicate, there isn’t necessarily a distinct time-point at which care transfers from primary to secondary care. There may be an ‘overlapping’ period of shared responsibility.

- *Date of diagnosis* – our study illustrates differing perceptions amongst patients and GPs, and these need to be recognised when interpreting responses. Patients appear to consistently view this date as the time they were made aware of their diagnosis – this may be substantially different to the date of histological confirmation.

## Conclusions

Measuring routes and time intervals in cancer diagnostic pathways is a complex task. In the past there has been little methodological rigour in studies reporting diagnostic intervals. While the Aarhus Statement has helped to standardise approaches, there are still significant challenges in developing methods of enquiry which elicit valid responses. This study highlights many of the areas where ED researchers need to be especially cautious in measuring time-points and interpreting responses. While the results of the study give an important steer on questionnaire wording, it should be acknowledged that measuring complex routes to diagnosis with self-completion questionnaires requires caution, and steps should be taken to increase clarity of responses wherever possible. A further option is to use more than one data source to enable ‘triangulation’ of responses – although this will likely entail further cost and burden. Our recommendations are designed to complement those in the Aarhus Statement, and should assist CDRs in developing improved survey instruments – and in interpreting the data those instruments generate.

## Abbreviations

A&E: Accident and emergency
CDR: Cancer diagnosis researcher
COPD: Chronic obstructive pulmonary disease
DC: Domenica Coxon (author)
ED: Early diagnosis
GP: General practitioner
SS: Suzanne Scott (author)
